# Optimising the Therapeutic Interval for Biologics in Patients with Psoriasis

**DOI:** 10.3390/life12122075

**Published:** 2022-12-10

**Authors:** Jose Manuel Dodero-Anillo, Inmaculada Concepcion Lozano-Cuadra, Esmeralda Rios-Sanchez, Maria Jose Pedrosa-Martinez, Jose Carlos Ruiz-Carrascosa, Manuel Galan-Gutierrez, Jose Carlos Armario-Hita

**Affiliations:** 1 Clinical Pharmacology, Hospital Universitario Puerto Real, 11510 Puerto Real, Spain; 2 Dermatology and Venorology, University of Cadiz, 11003 Cádiz, Spain; 3 Hospital Pharmacy, Hospital Universitario Puerto Real, 11510 Puerto Real, Spain; 4 Dermatology and Venereology, Hospital Universitario San Cecilio, 18016 Granada, Spain; 5 Dermatology and Venereology, Hospital Universitario Reina Sofia, 14004 Córdoba, Spain; 6 Dermatology and Venereology, Hospital Universitario Puerto Real, 11510 Puerto Real, Spain

**Keywords:** psoriasis, biologic, therapeutic interval, PASI, optimise, infliximab, adalimumab, etanercept, therapeutic drug monitoring

## Abstract

In our clinical experience, more than half of patients do not present a complete response to biologic drugs, or drug loses its efficacy over time. Plasma determinations of drug and anti-drug antibodies levels are an objective tool for optimisation in these patients; however, established therapeutic ranges are not suitable, so the objective of this study was to study these patients and optimise their healthcare. We have made a retrospective, observational study, using data of plasma levels of drugs and anti-drugs antibodies of infliximab, adalimumab or Etanercept, we summarise all data and make a study of sensitivity, specificity, positive and negative predictive value on current therapeutic ranges. We have found a statistically significant association between subtherapeutic levels and therapeutic failure in psoriasis treated with infliximab and adalimumab. New ranges were found with higher sensitivity than the established ones, we propose 2–10 µg/mL therapeutic range for infliximab, 3–11 µg/mL for adalimumab, and 1–7 µg/mL for etanercept. In conclusion, levels of drug and anti-drug antibodies are a decisive tool for predicting therapeutic response. The current therapeutic ranges may have minimum values that are excessively high, owing to which lowering them significantly increases the sensitivity of the test in all cases, and negative predictive value in the case of etanercept. Further prospective studies are needed to prove the usefulness of these new ranges.

## 1. Introduction

Biologic drugs have revolutionised therapeutics for autoimmune diseases (psoriasis, rheumatoid arthritis, inflammatory bowel disease, etc.,), but the truth that we can found in daily clinical practice is that more than half of the patients do not present a complete response to the drug, or the drug loses its efficacy over time [1].

The pathogenesis of psoriasis involves a process of exaggerated epidermal proliferation, in which keratinocytes take on a very high rate of multiplication, alongside a primarily lymphocytic chronic inflammatory infiltrate. Various studies point to a predominance of the Th1-type (cellular) immune response, wherein the presence of tumour necrosis factor α (TNF-α) triggers the activation of other molecules that perpetuate the process [2]. TNF-α can be produced by both keratinocytes and T4 lymphocytes. This substance is capable of activating dendritic cells (DC), auxiliary immune cells derived from the bone marrow that are present in epithelial and lymphatic tissues. From a structural point of view, they are characterised by their multiple membranous projections, from which they take their name. Their importance in psoriasis stems from their action as antigen-presenting cells (APCs) for undifferentiated T-lymphocytes. Both dendritic cells and macrophages synthesise two heterodimers that are important throughout this inflammatory process after stimulation with TNF-α: interleukin (IL)-12 and IL-23. This entire process is useful to understand how the biologic drugs that this work addresses work.

To evaluate the effectiveness of the treatment in psoriasis, the Psoriasis Area and Severity Index (PASI) is normally used [3]. PASI is usually used to classify intensity and extension of body surface area affected by psoriasis. In this index, the following items are evaluated: erythema, infiltration, and desquamation of plaque psoriasis in each corporal area, taking into account the surface of each area. In this study, we will use PASI 75 to evaluate the clinical condition of each patient, which means that there is a decrease of 75% compared to initial PASI [4].

Plasma determination of levels of drug and anti-drug antibodies are an objective tool that has been of great use in clinical practice. It can be used to establish close monitoring, and even when contemplating treatment optimisation in patients [5,6,7].

The drug’s pharmacokinetics are conditional on certain factors, such as: age, sex, serum albumin, molecular weight, comorbidities, underlying disease characteristics, concomitant immunosuppressant use, or the significant role played by the formation of anti-drug antibodies [7]. Based on the literature reviewed [8,9], we currently have a situation wherein the therapeutic ranges used do not correlate closely with the patient’s clinical response, leading us to question whether the therapeutic intervals are set correctly.

Based on this context, there is a need to improve the healthcare of patients with a diagnosis of severe psoriasis by contributing scientific evidence on the true efficacy of biologic therapies based on therapeutic drug levels.

To this end, a retrospective, observational study has been conducted to analyse clinical data in patients in treatment with different monitored biologic drugs—infliximab (IFX), adalimumab (ADL) and etanercept (ETN)—and levels of drug and anti-drug antibodies.

Infliximab is a chimeric monoclonal antibody IgG1 with high specificity against TNF-α that acts by blocking the interaction with its receptors. Its therapeutic regimen is a dose of 5 mg/kg at week 0, week 2, week 6 and then a maintenance dose every 8 weeks. With this regimen, it reaches a PASI75 in 67% of patients treated at week 12 [4]. Adalimumab is a human monoclonal antibody IgG1 also against TNF-α, such as infliximab [10]. Its therapeutic regimen is an initial dose of 80 mg at the first week and then 40 mg at alternate weeks, reaching a PASI75 in 53% of patients at week 12 and 80% at week 16. Etanercept is a human recombinant protein against TNF-α, both free fraction and bound to its receptor. Using 25–50 mg weekly PASI75 reaches 35% of patients at week 12, getting to 50% if the dose is doubled. Etanercept does not have rebound effect and lesion-free period after discontinuation is around 3 months [2].

Attending to the immune response to this biologic treatments, it seems to follow a temporal transitory pattern, different at the classic immune response to an exogenous protein, because, when the treatment is discontinued, anti-drug antibody even disappears [11]. However, not all anti-drug antibody act equally, some neutralise the drug and others attach to him, altering its pharmacokinetic or pharmacodynamics. Both situations affect the therapeutic ranges [12].

Therefore, in order to analyse these peculiarities of biological treatment, new therapeutic ranges have been proposed for these drugs which, in comparison with the established ranges, might allow the therapy to be optimised in these patients.

## 2. Materials and Methods

### 2.1. Patients

Data were collected from patients of the Dermatology Department of Hospital Universitario Puerto Real, which offers specialist healthcare in the Bahía de Cádiz—La Janda District (Andalusia, Spain). This centre covers a target population of 326,674 documented inhabitants, of whom, according to the Hospital Universitario Puerto Real’s Documentation and Information service, some 30,000 patients/year are treated in the Dermatology Unit. Plasma determinations of drug and anti-drug antibodies levels were collected from the records of patients at Clinical Pharmacology unit, in a retrospective way.

This study included adult patients with severe psoriasis vulgaris who were in the maintenance phases with any of the selected biologics: IFX, ADL, or ETN. The Andalusian Health System’s DIRAYA digital medical records system was used to collect the data.

We have included patients who has been treated with at least one of these drugs once, we did not analyse again patients who got retreated after a discontinuation of treatment due a to remission of psoriasis.

### 2.2. Variables of Interest

The following quantitative variables were collected: age, baseline PASI score, number of patients with a change in treatment dose, number of patients who responded (those who maintained a PASI75 response: 75% or greater reduction in PASI score from the baseline value) or did not respond (therapeutic failure) after treatment, and plasma levels of the drug and anti-drug antibodies. The qualitative variables collected were: sex, drug prescribed, drug plasma levels categorised based on the established therapeutic range (subtherapeutic, therapeutic and supratherapeutic), presence of anti-drug antibodies in plasma (yes or no) and maintenance of a PASI75 response after a change in dose (yes or no).

### 2.3. Accuracy of the Therapeutic Ranges

The established therapeutic ranges for IFX, ADL, and ETN were 3–10 µg/mL [13], 5–12 µg/mL [13], and 2–7 µg/mL [14], respectively. The new therapeutic ranges proposed based on the results were 2–10 µg/mL, 3–11 µg/mL, and 1–7 µg/mL.

For both the therapeutic and proposed ranges for IFX, ADL, and ETN, a study was performed of sensitivity (probability that a patient who responds to the biologic treatment will achieve drug plasma levels within the established ranges), specificity (probability that a patient who does not respond will have drug plasma levels outside the therapeutic ranges), positive predictive value (PPV; probability of responding adequately to treatment when having drug plasma levels within the established range), and negative predictive value (NPV; probability of not responding to the biologic treatment when having drug plasma levels outside the selected range). Table 1 defines the conditions for a patient to be considered a true or false positive or negative.

### 2.4. Statistical Analysis

The sample size was calculated for a comparison of means, using an effect size of d = 0.35, a suggested standard measure between the small and medium effect size, a statistical power of 0.95, an α error of 0.05 and an A/B ratio of 1. A sample size of 96 patients was obtained. The program G*PowerR, version 3.192 using Cadiz University net, Spain, available from the website of the University of Dusseldorf, was used.

Frequency tables (%) and graphs were used for the categorical variables, and frequency tables (case intervals), graphs and parameters such as mean and standard deviation, among others, for the numerical variables. For the bivariate analysis, first it was tested whether the variables fitted a normal distribution. Either a Shapiro–Wilk W test or a Kolmogorov–Smirnov test was performed.

For the qualitative variables, the chi-squared test was used. For the categorical variables, contingency tables were constructed to calculate the odds ratio, and a chi-squared test was used to compare independence.

A *p*-value of <0.05 (95% CI) was considered statistically significant. The IBM program SPSS Statistics 22 manufactured by Cadiz University, Spain was used to perform the calculation and obtain the data.

## 3. Results

### 3.1. Patients’ Demographic and Clinical Data

A total of 98 patients were included. The group was made up of 66.3% men and 33.7% women. The mean age of the group was 54.3 ± 12.9 years (21 to 84 years) and the duration of disease was 8.3 ± 2.1 years at the start of the study. To study weight, we have measured the body mass index (BMI) of each patient (mass (kg)/height (m)^2^). The mean BMI was 29.4 ± 5.1, with some 38.5% of patients being overweight, 23.1% morbidly obese, and just 7.6% severely obese. The most common comorbidities in this group included dyslipidaemia (41.7%), psoriatic arthritis (35.4%), diabetes mellitus (33.3%), and hypertension (27.1%). All of the patients had severe psoriasis with a PASI score above 10. Most of the patients included in the study were naïve to the treatment studied, and just 17.3% of the patients were resuming prior biologic treatments.

The patients included in the study represented a total of 141 biologic treatments, the most common of which was ETN (53.9%, 76 patients), followed by ADL (31.9%, 45 patients) and IFX (14.2%, 20 patients).

### 3.2. Response to Infliximab

In the IFX group, of the 20 patients treated, nine had subtherapeutic plasma levels, eight had therapeutic plasma levels and three has supratherapeutic plasma levels (Table 1). In addition, two of the patients with subtherapeutic plasma levels were positive for anti-drug antibodies. The association between the presence of subtherapeutic plasma levels and therapeutic failure was statistically significant (*p* < 0.001) (Table 1).

However, a third of patients with subtherapeutic levels of the drug continued to have a therapeutic response. Considering these patients as responding to treatment, the mean therapeutic plasma level of IFX would be 5.24 ± 3.80 µg/mL. Moreover, the study of plasma levels allowed the dose to be optimised in 15% of the patients without loss of effectiveness.

### 3.3. Response to Adalimumab

In the ADL group, 28 of the 45 patients treated had subtherapeutic plasma levels, while 17 had therapeutic levels (Table 1). Just two non-responding patients with subtherapeutic levels developed positive anti-drug antibodies. A statistically significant association was found between responding to treatment and having therapeutic or subtherapeutic drug plasma levels (*p* < 0.01) (Table 1).

A high number (18 out of 35; 51.4%) of responding patients with subtherapeutic levels, as a consequence of treatment optimisation by extending the interval between doses, was observed. Considering these patients as responding to treatment, the mean therapeutic plasma level of ADL would be 4.93 ± 2.78 µg/mL. Moreover, studying plasma levels allowed the dose to be optimised in 40% of cases.

### 3.4. Response to Etanercept

Of the 76 patients treated, 35 had subtherapeutic plasma levels, 39 had therapeutic plasma levels, and 2 had supratherapeutic plasma levels (Table 1). No patient developed positive anti-drug antibodies. A statistically significant association was found between having subtherapeutic plasma levels and responding to treatment (*p* < 0.03) (Table 1).

It is noteworthy that a high number (29 out of 54; 53.7%) of responding patients with subtherapeutic levels, as a consequence of treatment optimisation by extending the inter-dose interval, at least at six months of treatment, was observed. Considering these patients as responding to treatment, the mean therapeutic plasma level of ETN would be 2.61 ± 1.61 µg/mL. Moreover, studying plasma levels allowed the dose to be optimised in 39.47% of cases.

### 3.5. New Therapeutic Ranges

With the new proposed therapeutic ranges, an increase was observed in patients responding within range versus those out-of-range for the three drugs studied: 45% to 55% for IFX, 38% to 58% for ADL, and 30% to 64% for ETN (Table 2).

For IFX, sensitivity increased in a statistically significant manner, from 64% to 78% (*p* = 0.02) with the new proposed range versus the established range. The difference was not significant for the NPV and there were no changes in specificity and PPV (Table 3).

Similarly, for ADL, sensitivity increased in a statistically significant manner, from 48% to 74% (*p* < 0.001) with the new proposed range versus the established range. The differences in specificity, PPV and NPV were not significant (Table 3).

For ETN, statistically significant increases in sensitivity (59% to 72%; *p* < 0.0001) and PPV (46% to 91%; *p* = 0.08) were observed with the proposed range versus the established range, while the changes in specificity and NPV were not significant (Table 3).

## 4. Discussion

In this study we evaluated the response outcomes obtained in psoriasis patients for each type of biologic therapy, IFX, ADL and ETN, based on their therapeutic ranges. However, the scant literature relating to the subject studied in this work considerably limited comparisons of our results.

For IFX, we found a statistically significant association between the presence of subtherapeutic plasma levels and therapeutic failure, as well as how the appearance of anti-drug antibodies was always predictive of therapeutic failure. These results are consistent with those published in previous studies, which support the same hypothesis linking drug plasma levels and the appearance of anti-drug antibodies with therapeutic response or failure [15,16]. Some 10% of our patients developed antibodies to IFX, which is within the range published in the literature of 5.4% to 43.6% [17,18,19,20,21]. We calculate that studying plasma levels of IFX and antibodies thereto allowed us to optimise the dose in 15% of the patients without a loss of effectiveness.

With regard to ETN, we found a statistically significant association between subtherapeutic plasma levels and responding to treatment. The number of patients who responded to treatment with subtherapeutic levels was surprisingly high (29 of the 76 studied), a consequence of treatment having been optimised by extending the interval when the patient had therapeutic drug levels and a good clinical course based on a physician’s assessment. This led us to think that we have been using a range of levels that leaves out a large number of responding patients, and suggests that the range should be adjusted.

It is less common for anti-drug antibodies to develop in patients treated with ETN [22,23]. Current literature has determined that between 0% and 18.3% of patients with psoriasis in treatment with ETN develop anti-drug antibodies [24,25,26,27,28,29] and, in fact, no patient had developed them in our study. Moreover, studying plasma levels allowed us to optimise the dose in 39.47% of cases.

Lastly, when we studied ADL, we found a statistically significant association between responding to treatment and having therapeutic or subtherapeutic drug plasma levels, with 18 of the 45 patients presenting a PASI75 response with subtherapeutic levels and 17 with therapeutic levels. Once again, we observed that a large number of patients who responded had subtherapeutic levels, at 51%. These findings support the proposal to adjust the range of plasma levels for the biologics studied. Just 4.5% of our patients presented anti-ADL antibodies, lower than the mean published in the literature, which places the percentage of patients with psoriasis treated with ADL who develop anti-drug antibodies between 6.5% and 45% [26,27,28,29,30,31]. Moreover, studying plasma levels allowed us to optimise the dose in 40% of cases.

Given the significant number of patients who respond with subtherapeutic levels in the population we studied, we questioned whether these ranges were well-established or whether we could adjust them to more accurately predict the therapeutic response. Accordingly, we decided to calculate sensitivity, specificity, PPV and NPV for both the current established range of each biologic drug and the new range proposed based on the results of our study.

For the drug IFX, the manufacturer’s established range of plasma levels is 3–10 µg/mL. However, in our study, we propose a new therapeutic range (2–10 µg/mL) that would significantly improve the sensitivity of the test when predicting which patients will respond. These results are in keeping with that published by Takahashi et al. [16], who called attention to IFX plasma values of 0.92 µg/mL, i.e., lower than the therapeutic range proposed on commercial kits, as a potential minimum concentration to obtain a good clinical response (PASI > 75). Finding the lowest drug plasma concentration with a therapeutic response in our patients is a priority if we also assume that, in patients with severe psoriasis treated with IFX, continuous therapy is preferable over intermittent therapy, as was demonstrated by the Reich et al. [31] ’s work on 222 patients.

With regard to ADL, the manufacturer’s established therapeutic range is 5–12 µg/mL. However, our study showed that the mean plasma levels associated with a therapeutic response in our group of patients was 4.93 µg/mL, which led us to propose a new interval (3–11 µg/mL). In this sense, it is worth noting that the various published studies with the objective of defining the minimum plasma levels associated with PASI75 have shown a downward trend in recent years. Thus, while in the first work by Lecluse et al. (2010) [28] this was 9.7 µg/mL, another study placed it at 7.84 µg/mL [27], and more recently in 2015, the estimated range was 3.51–7.00 µg/mL in patients with psoriasis who responded to treatment [25]. This last figure is much closer to the interval we have proposed based on the results of our work. The new proposed range (3–11 µg/mL) is associated with a significant improvement in sensitivity, owing to which is more likely that a patient with a good therapeutic response will fall within our new interval.

Lastly, with regard to ETN, the manufacturer’s established range of plasma levels is 2–7 µg/mL. However, our new proposed range (1–7 µg/mL) allows a significant improvement in both sensitivity and NPV. We found data in the literature that refer to this fact, but taking into account the high percentage of optimised patient who maintain a PASI75 therapeutic response, as well as the data obtained in our study, it seems evident that the new proposed range more accurately predicts therapeutic response based on drug plasma levels.

To stablish limits to the analysis of our data we have to point out that it is known that efficacy of treatment with anti-TNF-α may be reduced at obese population, and we have 7.6% of obese patients, due to this probable limitation, dose was adjusted by the hospital pharmacy unit to try to reach the maximum effectiveness, however this can be a limitation of the results interpretation.

## 5. Conclusions

Drug and anti-drug antibody levels are a useful tool for predicting therapeutic response and establishing biologic therapy optimisation in patients with severe psoriasis.

We have found with the results of our study that the officially established ranges may be excessively high, according to our population, such that adjusting them downwards significantly improves the sensitivity of this test in all cases, as well as NPV for ETN in the population that we have studied.

As a result of these findings, wherein a considerable percentage of patients were out of range and paradoxically they have a good clinical condition. However, this proposal of new therapeutic ranges should be backed up with more prospective clinical studies in routine practice to reach wider consideration.

## Figures and Tables

**Table 1:** Distribution of patients based on whether they responded (PASI75) or did not respond (No PASI75) to the drugs by plasma levels.

Drugs and Plasma Levels	PASI75	No PASI75	*p* Value	OR
**Infliximab**				
Therapeutic	8	0	0.01	N/A *
Subtherapeutic	3	6	<0.001	N/A
Supratherapeutic	3	0	0.01	N/A
**Adalimumab**				
Therapeutic	17	0	0.005	N/A
Subtherapeutic	18	10	0.005	N/A
Supratherapeutic	0	0	N/A	N/A
**Etanercept**				
Therapeutic	23	16	0.01	0.29 (0.09–0.829)
Subtherapeutic	29	6	0.03	3.09 (1.05–9.10)
Supratherapeutic	2	0	N/S *	N/A

* N/A: not available; N/S: not significant.

**Table 2:** Number of patients who responded (PASI75) or did not respond (No PASI75) to the drugs by whether they fall within the established and proposed therapeutic ranges.

Drugs and Therapeutic Ranges	Patients Who Responded			
	with Established Ranges		with Proposed Ranges	
	PASI75	No PASI75	PASI75	No PASI75
**Infliximab**				
In range	9 (45%)	0	11 (55%)	0
Out of range	5 (25%)	6 (30%)	3 (15%)	6 (30%)
**Adalimumab**				
In range	17 (38%)	2 (4%)	26 (58%)	4 (9%)
Out of range	18 (40%)	8 (18%)	9 (20%)	6 (13%)
**Etanercept**				
In range	23 (30%)	16 (21%)	49 (64%)	19 (25%)
Out of range	31 (41%)	6 (8%)	5 (7%)	3 (4%)

**Table 3:** Analysis of sensitivity, specificity, positive predictive value (PPV) and negative predictive value (NPV) for the drugs in the established and proposed ranges.

Drugs and Parameters	Values		*p* Value
	with Established Ranges	with Proposed Ranges	
**Infliximab**			
Sensitivity	64%	78%	0.02
Specificity	100%	100%	N/S *
PPV	100%	100%	N/S
NPV	55%	67%	N/S
**Adalimumab**			
Sensitivity	48%	74%	<0.001
Specificity	80%	60%	N/S
PPV	89%	87%	N/S
NPV	30%	40%	N/S
**Etanercept**			
Sensitivity	59%	72%	<0.0001
Specificity	16%	38%	N/S
PPV	46%	91%	0.008
NPV	28%	14%	N/S

* N/S: not significant.
